# Discarded diversity: novel megaphages, auxiliary metabolic genes, and virally encoded CRISPR-Cas systems in landfills

**DOI:** 10.1186/s12985-025-02990-6

**Published:** 2025-11-11

**Authors:** Nikhil A. George, Zhichao Zhou, Karthik Anantharaman, Laura A. Hug

**Affiliations:** 1 Department of Biology, University of Waterloo, Waterloo, ON Canada; 2 Department of Bacteriology, University of Wisconsin – Madison, Madison, WI USA

**Keywords:** Phage, Viruses, CRISPR, CRISPR-Cas, Landfill, Municipal solid waste

## Abstract

**Background:**

Viruses are the most abundant microbial entities on the planet, impacting microbial community structure and ecosystem services. Despite outnumbering bacteria and archaea by an order of magnitude, viruses have been comparatively underrepresented in reference databases. Metagenomic examinations have illustrated that viruses of bacteria and archaea have been specifically understudied in engineered environments. Here we employed metagenomic and computational biology methods to examine the diversity, host interactions, and genetic systems of viruses predicted from 27 samples taken from three municipal landfills across North America.

**Results:**

We identified numerous viruses that are not represented in reference databases, including the third largest bacteriophage genome identified to date (~ 678 kbp), and noted a large diversity of viruses in landfills that has limited overlap across landfills and is distinct from viromes in other systems. Host-virus interactions were examined via host CRISPR spacer to viral protospacer mapping which captured hyper-targeted viral populations and six viral populations predicted to infect hosts across multiple phyla. Auxiliary metabolic genes (AMGs) were identified with the potential to augment hosts’ methane, sulfur, and contaminant degradation metabolisms, including AMGs not previously reported in the literature. CRISPR arrays and CRISPR-Cas systems were identified from predicted viral genomes, including the two largest bacteriophage genomes to contain these genetic features. Some virally encoded Cas effector-like proteins appear distinct relative to previously reported Cas effectors and are interesting targets for potential genome editing tools.

**Conclusions:**

Our observations indicate landfills, as heterogeneous contaminated sites with unique selective pressures, are key locations for diverse viruses and atypical virus-host dynamics.

**Supplementary Information:**

The online version contains supplementary material available at 10.1186/s12985-025-02990-6.

## Background

In one of the largest examinations of Earth’s virosphere to date, it was noted that samples from engineered sites have poorly characterized viral diversity [1]. Two recent studies of wastewater treatment plants confirmed this, with > 98% of detected viral contigs unmatched to a representative subset of the IMG/VR database in one [2] and > 99% unmatched to the NCBI virus database in the other [3]. Landfills, similar to wastewater treatment plants, are highly engineered, polluted and environmentally significant systems [4–6]. In municipal waste systems, we previously predicted that viruses of bacteria and archaea have diverse infection dynamics with their hosts based on CRISPR spacer to virus protospacer matching [7]. The open questions remain of whether the notable virus-host interactions observed in a single municipal waste site, such as hyper-targeted viral elements, interviral conflicts, and putative cross-phylum-infecting phages [7], are consistent across other municipal waste sites.

Bacteriophages are predicted to be host specific, but recent studies have suggested the presence of phages with diverse host ranges in different environments. In research on both isolated phages and those identified in silico, bacteriophages have been shown or predicted to infect across distinct bacterial phyla [1, 7–10] and domains of life [2, 10]. While the mechanisms of viral infection across organisms from different phyla or domains are likely very complex and difficult to resolve without wet-lab experiments, the potential presence of such viruses with large host ranges and their isolation can be important for biotechnological applications including phage therapy. There are multiple computational methods to predict microbial hosts of viruses [11, 12], with CRISPR spacer to viral protospacer matching having relatively low sensitivity, but high specificity [11].

Viruses of bacteria and archaea have genomes that are variable in size, but are canonically considered short, efficient genomic elements constrained by their requirement to package into capsids for infection [13]. Recently, genomic approaches have identified an increasing number of phage genomes ≥ 200 kbp in size, where phages with genomes ≥ 200 kbp are referred to as jumbo phages and phages with genomes ≥ 500 kbp are referred to as megaphages [1, 14–16]. The current record length for a circularized megaphage genome is 735 kbp [17]. These less-streamlined phage genomes have more coding space, and have been shown to carry tRNAs and associated enzyme-coding genes, other translation machinery, auxiliary metabolic genes (AMGs), and CRISPR-Cas systems [17].

During infections, viruses may augment or modify the metabolism of their hosts through AMGs [18–22], including provision of genes for contaminant modification and degradation. Recent research has identified viruses encoding arsenic resistance and transporter genes in arsenic-contaminated paddy soils [23], and showed viruses aiding their hosts’ survival in organochlorine contaminated soils through AMGs involved in organochlorine degradation [24, 25]. This phenomenon of contaminant modification/degradation genes being provisioned by viruses to their hosts has yet to be examined in municipal waste sites, which are highly and heterogeneously contaminated systems.

Despite the widespread adoption of CRISPR-Cas-based methods for genome editing and other biotechnological tools in both academic and industrial labs, the technology still has limitations [26]. Discovery of new, divergent Cas proteins and CRISPR-Cas system assemblages is of interest to address these limitations, in hopes of broadening the current application space [26]. Virus-encoded CRISPR arrays and CRISPR-Cas systems have been identified from a variety of environments [7, 17, 27–34] and are involved in crippling host viral defense systems [29]; regulating host transcription and translation [17]; CRISPR-Cas system inhibition [32]; and interviral conflicts [7, 32, 33]. Recent discoveries of some of the most streamlined CRISPR-Cas systems from viruses [17, 34, 35] established viruses as a potential hot spot for new CRISPR-Cas systems with biotechnological value.

Here we examine the viral diversity within three municipal landfills distributed across North America, examining host-virus dynamics, CRISPR spacer conservation across geographic distance, and the unique diversity of the landfill virome. We identify new lineages within the jumbo phage and megaphage viral pantheon and characterize AMGs contributing to the landfill microbial community. Finally, we expand the examination of virally encoded CRISPR-Cas systems to municipal landfill microbial communities, identifying several streamlined CRISPR-Cas-like systems as potential targets for developing efficient biotechnological tools.

## Results

### Sampling sites and metagenomes

This study makes use of 27 metagenomes that were sequenced from samples from three distinct landfills. Fourteen metagenomes were sequenced from an active municipal landfill and adjacent aquifer in Southern Ontario (SO), sampled twice one year apart (July 2016 [SO_2016] and October 2017 [SO_2017]) and described previously [7, 36]. Nine metagenomes were from a northeastern United States active municipal landfill (NEUS) in which nine distinct cells, units in which waste is held within the landfill, were sampled in February 2019 as described previously in an examination of the methane cycling microbial community [37]. At the time of sampling, the oldest cell had received waste from 1980 to 1982 (39 years of waste decomposition time), whereas the youngest cell began receiving waste in 2014 (5 years of waste decomposition time). The final four metagenomes came from a closed landfill from Southern California that was sampled in June of 2019 (CA_2019). The Californian landfill was operational from the mid 1960s to the mid 1990s and received both municipal and hazardous waste. The landfill has been closed for over 20 years but remains under management for contaminant containment. From the three landfill sites, samples were taken from leachate wells, leachate collection cisterns, leachate-impacted groundwater wells, and, in one case, a leachate treatment facility (CA landfill, influent and biosolids fraction sampled) (Table S1). The viral component of the SO landfill metagenomes was previously described solely for viral-host interactions [7]. None of the other metagenomes have been analyzed for their viral fraction, and the CA_2019 landfill samples have not previously been reported. Metagenome statistics for all sites are reported in Table 1.

### Viral proportion and genome sizes

**Fig. 1:**
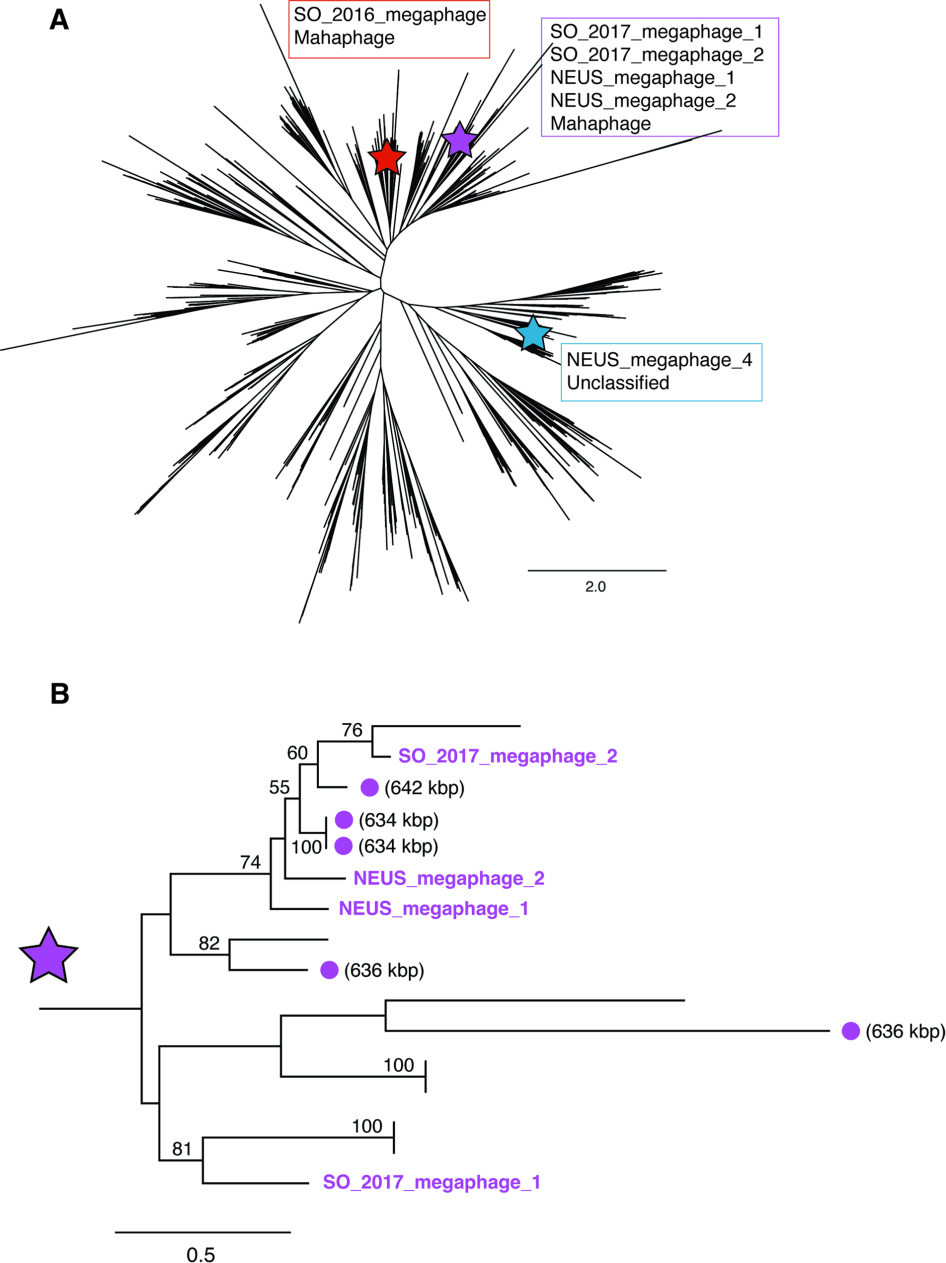
Landfill-derived megaphages are unique but fall within known megaphage diversity. **A**: Phylogenetic placement of newly discovered megaphages. Phylogenetic reconstruction was based on an alignment of large terminase subunit encoding genes from this study (*n* = 6) and those from a reference study (*n* = 1,256 [17]). Stars indicate the clades in which megaphages identified in this study were placed [17]. **B**: Placement of four newly discovered megaphages in a clade specific to huge phages. References phages in this clade include some of the largest phages identified from a previous study [17]. The sizes of reference phage genomes are indicated if they are ≥ 600 kbp. Bootstrap support is noted when above 50

Viral scaffold prediction was conducted by VirSorter2 (VS2) [38]. Viral genome quality was assessed with CheckV [39], followed by clustering with CD-HIT for all sequences above 5 kb in length [40], which resulted in a total of 22,658 unique viral scaffolds in the SO_ 2016 samples (27 scaffolds ≥ 200 kbp). The SO_2017 samples had a total of 37,182 unique viral scaffolds (69 scaffolds ≥ 200 kbp), the NEUS samples had a total of 31,944 unique viral scaffolds (31 scaffolds ≥ 200 kbp), and the CA_2019 samples had a total of 5,419 unique viral scaffolds (11 scaffolds ≥ 200 kbp). Collectively, 97,203 viral scaffolds ≥ 5 kbp were identified. Notably, seven of these viral scaffolds were predicted to be nearly complete or complete megaphage genomes. Six out of seven of these sequences were predicted to be 100% complete according to CheckV’s identification of high-confidence Direct Terminal Repeats (DTRs) in the sequences, and all seven sequences were predicted to circularize by vRhyme, and circularization confirmed with read mapping (Table 2 [41]). Large terminase subunit genes, genes encoding part of the viral DNA packaging machinery, were identified in six out of seven putative megaphages. Five of these sequences placed within the Mahaphage clade of giant phages [17] in a phylogenetic tree (Fig. 1), and one of them placed within an unclassified lineage under the most recent classification scheme for giant phages [17]. The two largest putative megaphage sequences were ~ 641 kbp (NEUS_megaphage_1) and ~ 678 kbp (SO_2017_megaphage_1) in size. The 678 kbp phage is, to our knowledge, the third largest phage genome reported to date, with the largest previously reported phage genomes being 634, 636, 642, and 735 kbp [17], 656 kbp [42], 660kbp [43], and 717 kbp [44].

**Table 1:** Summary statistics for metagenomes, metagenome-assembled genomes (MAGs), viral scaffolds, and viral metagenome-assembled genomes (vMAGs) from the sampling sites. Scaffolds within vMAGs are included in the viral scaffold counts

Site	# of Metagenomes	Read count	Scaffolds ≥ 5 kbp	MAGs^1^	Viral scaffolds ≥ 5 kbp^2^	vMAGs^3^	% viral^4^
SO_2016	6	1,135,023,824	190,754	688	23,893	3,440	12.43
SO_2017	8	1,822,382,124	269,004	1,064	39,612	5,268	13.53
NEUS_2019	9	1,869,347,618	530,926	1,881	36,453	4,199	6.66
CA_2019	4	862,249,102	231,655	762	6,410	829	2.15

^1^ Quality filtered bacterial and archaeal bins with > 70% completion, < 10% contamination

^2^ Viral scaffolds detected (prior to CD-HIT clustering)

^3^ Quality filtered viral bins encoding ≤ 5 redundant proteins

^4^ % of total nucleotides in assembled scaffolds ≥ 5 kbp predicted to be of viral origin

**Table 2:** Genome statistics for complete megaphages identified in this study

Megaphage	Genome length (bp)	Sample site	Gene count	VIBRANT AMG count	CRISPR-encoding (subtype, total spacer count)
NEUS_megaphage_4	508,676	NEUS	643	21	Y (unknown, 32)
SO_2016_megaphage	518,809	SO_2016	812	20	N
NEUS_megaphage_3	589,338	NEUS	791	17	N
SO_2017_megaphage_2	617,251	SO_2017	900	11	N
NEUS_megaphage_2	623,658	NEUS	920	10	N
NEUS_megaphage_1	641,243	NEUS	1,001	14	Y (unknown, 3)
SO_2017_megaphage_1	678,337	SO_2017	1,128	20	Y (II-B, unknown, 10)

### Diversity and taxonomy of landfill viruses

We were interested in whether landfill viral elements were conserved across landfill sites, or across ecosystems. We first used Vclust [45] to determine the extent of relatedness between viral elements in our study and those from related and disparate ecosystems as classified in IMG/VR v4 [46]. Inspired by a recent study examining prokaryotic and viral communities from anaerobic digesters [47], environments with anaerobic conditions and biogas production similar to landfills, we included viruses from these engineered systems in our comparison to landfill viruses. We clustered our 106,368 ≥ 5 kbp landfill viral elements with 100,000 ≥ 5 kbp viral elements from anaerobic digesters, groundwater, and wastewater, respectively (each obtained from IMG/VR v4 [46]), and 100,000 ≥ 5 kbp viral elements sampled at random from IMG/VR v4, for a total of 400,000 reference viral elements. The 100,000 viral elements randomly sampled from IMG/VR v4 encompassed engineered, environmental, and host-associated environments, ranging from bioreactors to the human respiratory system; however, ~ 73% of these viral elements came from aquatic environments, consistent with 75% of the viruses in IMG/VR v4 being from aquatic environments. Only 14,701/106,368 of our landfill viral elements (~ 13.8%) clustered with any of the groundwater, wastewater, anaerobic digester, or sampled IMG/VR v4 viral elements.

We next used Vclust to examine how similar viral communities were between the landfill environments. From this, 13,007/23,892 SO_2016 viruses (~ 54%), 26,631/39,612 SO_2017 viruses (~ 67%), 30,160/36,453 NEUS_2019 viruses (~ 83%), and 6,092/6,410 viruses (95%) did not cluster with any viral elements from the other landfills. Using geNomad [48], 33,609 (~ 97%) of our ≥ 10 kbp viral elements were classified to the class level as Caudoviricetes and only 573 (~ 1.7%) were classified to either the order level or family level.

### Host-virus connections within and across landfills

To identify the hosts of viruses, we first reconstructed metagenome-assembled genomes for all landfill datasets (see Methods). Next, we examined matches between CRISPR spacers extracted from CRISPR arrays detected in bacterial and archaeal MAGs to viral protospacers, both within a particular landfill site and across distinct landfills/times. SO_2016 showed the strongest targeting to NEUS_2019, having more spacer to protospacer matches than NEUS_2019 did to SO_2016, 147 vs. 76, despite NEUS_2019 having almost four times the number of spacers as SO_2016 (Table 3). SO_2017 and NEUS_2019 spacers targeted viruses in their own respective datasets most strongly. Despite having, at minimum, nearly four times fewer viral elements than any other dataset, CA_2019’s spacer to protospacer matches was the second highest out of all 16 comparisons (516 connections) and the highest proportionally (5.5%, Table 3).

All sample sites showed instances of hyper-targeted viral elements and/or vMAGs, which we previously defined as viral elements (or vMAGs) targeted at least 20 times by a single host’s complement of CRISPR spacers [7]. The maximum numbers of spacer-to-protospacer matches between a host MAG and a viral element (or vMAG) per dataset are 55 (SO_2016), 24 (SO_2017), 32 (CA_2019), and 22 (NEUS_2019). After curation, across all networks, a total of five vMAGs and one viral element were predicted to infect across distinct phyla (Table S2), with one vMAG from NEUS_2019 predicted to infect hosts from three distinct bacterial phyla: Bacteroidota, Cloacimonadota, and Firmicutes_B. The SO_2016 network was the only network that didn’t include putative cross-phylum infections. Considering the relatively low viral scaffold count from the CA_2019 dataset (Table 3), it showed relatively high host and viral interconnectivity in its network (Table 4). No megaphage hosts were determined from this survey. Three jumbo phages were confidently connected to one host MAG each, which were affiliated with the Flavobacteriales (*n* = 2, CA_2019) and the Fibrobacterales (*n* = 1, SO_2016). Each jumbo phage was connected to its respective host MAG by a minimum of seven spacer-protospacer matches.

**Table 3:** Host CRISPR spacer to viral protospacer targeting within and across municipal waste sites as counts (percent) spacers matching

Host targeting (CRISPR spacer count)	# of spacer-protospacer matches (# VE)			
	to SO_2016 (22,659 VE)	to SO_2017 (37,184 VE)	to NEUS_2019 (31,944 VE)	to CA_2019 (5,419 VE)
SO_2016 (5,446)	138 (2.5%)	75 (1.4%)	147 (2.7%)	10 (0.2%)
SO_2017 (12,578)	232 (1.8%)	248 (2.0%)	204 (1.6%)	13 (0.1%)
NEUS_2019 (20,236)	76 (0.4%)	297 (1.5%)	697 (3.4%)	45 (0.2%)
CA_2019 (9,299)	81 (0.9%)	15 (0.2%)	38 (0.4%)	516 (5.5%)

Viral elements were dereplicated for this analysis. VE = viral elements

**Table 4:** Virus-host networks summary

Network	Host MAGs	Viral scaffolds or vMAGs	Unique links
SO_2016	13	20	22
SO_2017	73	100	115
NEUS_2019	169	206	321
CA_2019	68	86	127

Virus-host interactions are based on host MAG CRISPR spacers and vMAG or viral element (depending on if element was binned) protospacer connections. Unique links = 1 MAG to 1 viral element, regardless of number of spacer-protospacer connections

### CRISPR spacer conservation across waste sites

To assess the conservation of CRISPR-Cas-based viral defenses across landfills, we next examined conservation of specific CRISPR spacers within and between landfills (Table 5). Generally, SO_2016 and SO_2017 had the most spacers in common, which was expected given they are temporal samples from the same location, with overlapping sampling sites. The CA_2019 spacer set had very limited overlap with the other landfill sites.

**Table 5:** Shared CRISPR spacers between landfill datasets

Dataset	Number of host spacers	Spacers shared with SO_2016	Spacers shared with SO_2017	Spacers shared with NEUS_2019	Spacers shared with CA_2019
SO_2016	5,361	N/A	1,252 (14.58%)	162 (1.41%)	6 (0.1%)
SO_2017	11,812	1,252 (14.58%)	N/A	174 (1.18%)	10 (0.11%)
NEUS_2019	17,581	162 (1.41%)	174 (1.18%)	N/A	17 (0.14%)
CA_2019	7,223	6 (0.1%)	10 (0.11%)	17 (0.14%)	N/A

Each dataset’s host spacers were dereplicated with CD-Hit at 100% identity for this analysis. Shared spacers required exact matches. Reverse complement matches were included in this analysis. Cases where both a query spacer and its reverse complement had exact matches in the target database were considered as a single match. % shared was calculated by dividing the number of spacers shared between samples by the total number of spacers across both samples.

### Viral auxiliary metabolic genes

**Fig. 2:**
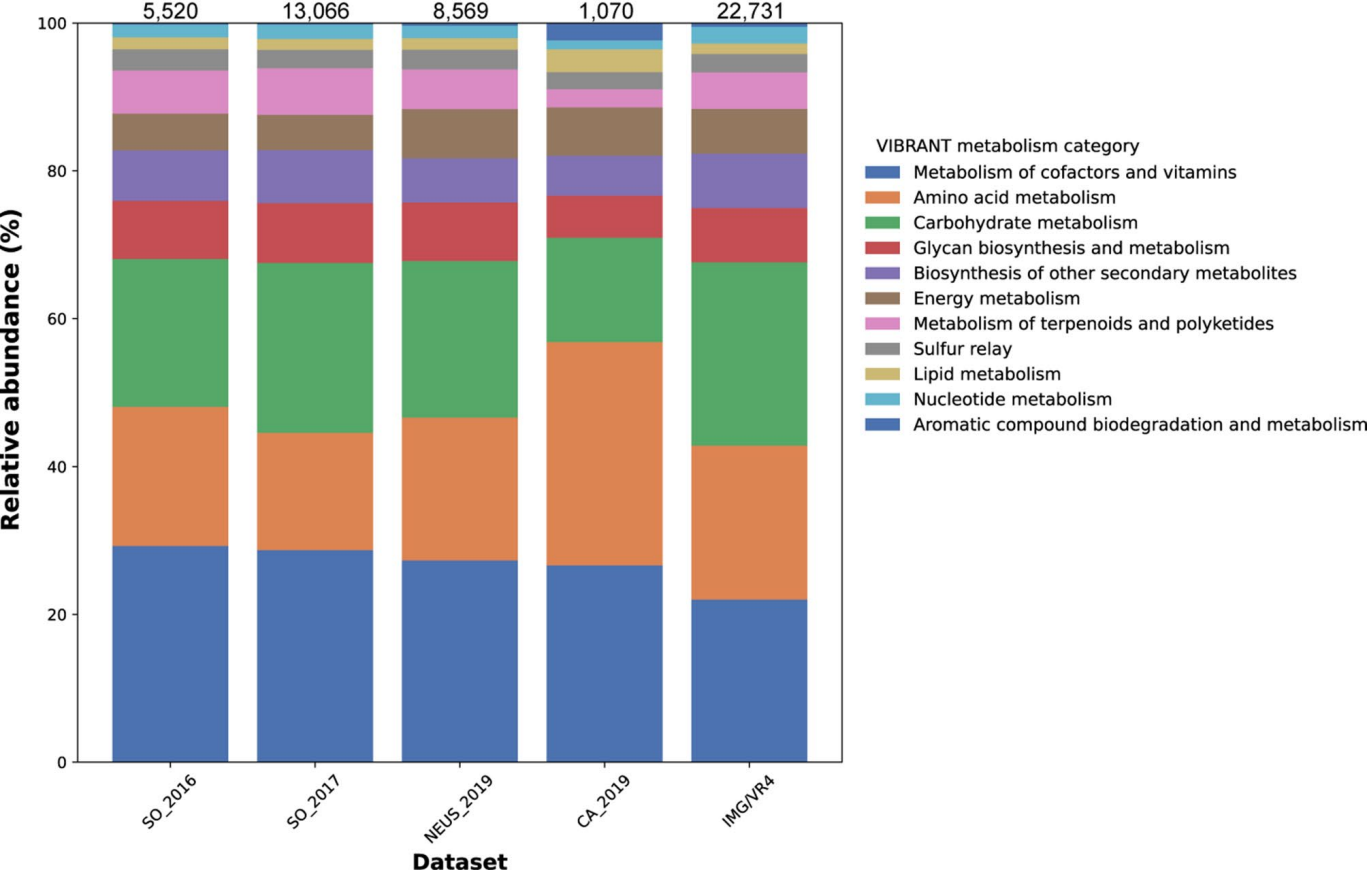
AMG metabolism category proportions across datasets. AMGs were detected using VIBRANT [79]. The IMG/VR dataset was used as a reference and was generated by randomly sampling 100,000 viruses from IMG/VR4 that were ≥ 5 kbp in size. The legend denotes AMG metabolism categories assigned by VIBRANT. Counts of viral elements ≥ 5 kbp included in the AMG search are as follows: SO_2016–23,893, SO_2017–39,612, NEUS_2019–36,453, CA_2019–6,410. Counts of predicted AMGs per dataset are reported at the top of each bar. 22,731 AMGs were predicted from the IMG/VR dataset, some of which may not have been previously reported

We next assessed the contribution from viruses to community metabolic processes by predicting AMGs encoded on viral elements. Across all landfills, a total of 581 unique AMGs were predicted (Table S3), representing functions across all major cellular processes (Fig. 2). AMG profiles between different landfills and a representative IMG/VR dataset were quite similar, except for the CA_2019 AMGs (Fig. 2). CA_2019 had a relatively high proportion of putative AMGs associated with lipid, amino acid, and aromatic compound metabolism, and a comparatively low proportion of AMGs associated with carbohydrate and terpenoids/polyketide metabolism. AMGs for processes that had not previously been associated with viruses or those that had very limited representation in literature were further manually curated (see Methods). AMGs of interest that passed manual curation included genes predicted to be involved in carbohydrate metabolism, methane metabolism, and sulfur metabolism (Table 6). The CA_2019 dataset included an AMG predicted to have a role in degradation of organochlorine compounds, which has been observed in previous studies [24, 25]. Numerous putative AMGs were also predicted in the putative megaphages - full details of VIBRANT-predicted AMGs encoded by these putative megaphages are available in Table S4.

**Table 6:** Notable putative auxiliary metabolic genes encoded by viruses in the landfill datasets

AMG name	Sites: # of instances per site	AMG function
*aprB*; adenylylsulfate reductase, subunit B [EC:1.8.99.2]	SO_2017: 1	Sulfur metabolism
*dehH*; haloacetate dehalogenase [EC:3.8.1.3]	CA:1	Chlorocyclohexane and chlorobenzene degradation, Chloroalkane and chloroalkene degradation
^E3.2.1.1 A; alpha-amylase [EC:3.2.1.1]	NEUS: 1	Polysaccharide metabolism
*^frhB*; coenzyme F420 hydrogenase subunit beta [EC:1.12.98.1]	SO_2016: 1 SO_2017: 8	Methane metabolism, Redox reactions
*hdrA2*; heterodisulfide reductase subunit A2 [EC:1.8.7.3 1.8.98.4 1.8.98.5 1.8.98.6]/4Fe-4 S binding domain	NEUS: 1 SO_2017: 1	Methanogenesis
*mcrD*; methyl-coenzyme M reductase subunit D	CA:1	Methanogenesis, anaerobic methane metabolism
*mddA*; methanethiol S-methyltransferase [EC:2.1.1.334]	CA:1 SO_2017:1	Methionine metabolism to dimethyl sulfide
*phoD; alkaline phosphatase D [EC:3.1.3.1]	CA:3 NEUS:1	Alkaline phosphatase
*^*rbcL*; ribulose-bisphosphate carboxylase large chain [EC:4.1.1.39]	NEUS:1 SO_2016:1 SO_2017:3	D-ribulose 1,5-bisphosphate carboxylation, carbon dioxide fixation
*soxX*; L-cysteine S-thiosulfotransferase [EC:2.8.5.2]	CA:1	Thiosulfate oxidation

Entries marked with an * represent functions also observed in 100,000 randomly selected IMG/VR prokaryotic viruses, but which are not widely reported in the literature. Functions are presented in alphabetical order according to AMG name. Highest confidence AMGs are marked with an ^ and were explored further with phylogenetic analyses

The highest confidence AMGs from Table 6 were alpha-amylase, *frhB*, and *rbcL*. Phylogenetic trees were used to place the alpha-amylase, *frhB*, and *rbcL* AMGs with their closest matches from Archaea and Bacteria on protein trees including well characterized reference sequences. The alpha amylase was near-identical to a hypothetical protein from a Prolixibacteriaceae bacterium within a cluster of glycoside hydrolase family 13 alpha amylase proteins, strengthening the prediction of its role in polysaccharide metabolism (Figure S1). The predicted protein products of the four high-confidence *frhB* genes represented two different sequence types, both clustering within a clade of bacterial coenzyme F420 reducing hydrogenase beta subunits, indicating a likely role in redox cycling (Figure S2). The RbcL RuBisCO subunit clustered with a group of “RbcL-like” proteins, and was distinct from a previously reported virally encoded RbcL (Figure S3) [49].

### Virally encoded CRISPR arrays

A total of 106,368 landfill-derived viral elements that were ≥ 5 kbp were included in examinations of virally encoded CRISPR arrays and novel Cas protein identification. Viral elements were not clustered with CD-HIT in this analysis to preserve differences in CRISPR array structure. 0.43% (454) of these viral elements encoded CRISPR arrays, a set which had an average length of ~ 47 kbp. Notably, the four longest CRISPR arrays were encoded on relatively short scaffolds (5,027 − 7,697 bp) that did not contain any detectable viral genes according to CheckV, or any detectable host (bacterial/archaeal) genes. These four scaffolds were conservatively considered false positive viral elements and were removed from subsequent analyses. Following this, 9/450 CRISPR array-encoding viruses were targeted in the virus-host networks, by predicted hosts from seven different phyla. Viral arrays encoded 2–67 spacers with an average spacer count of 8, with 11 viral arrays encoding at least 30 spacers.

The longest putative viral elements that encoded CRISPR arrays containing 30 + spacers included a 364 kbp jumbo phage (55 spacers) and a 508 kbp megaphage (32 spacers), both of which are predicted to be complete viral genomes according to CheckV. Multiple CRISPR arrays were less common, with 48 viral elements predicted to encode two or more CRISPR arrays (10.7% of CRISPR-array-encoding viral elements), with a maximum of six predicted CRISPR arrays for a single element. In total, 515 CRISPR arrays were associated with the 450 array-encoding viral elements. Spacers from these viral CRISPR arrays matched to 138 viruses that did not encode CRISPR arrays. We annotated hits using HMMs from the Virus Orthologous Groups Database (VOGDB) release 226 (https://vogdb.org/vogdb/226). The top hit was to a hypothetical protein. The top 10 hits included hypothetical proteins, collagen-related proteins (e.g., “REFSEQ Collagen triple helix repeat protein”), minor tail proteins, and tail fiber proteins.

**Fig. 3:**
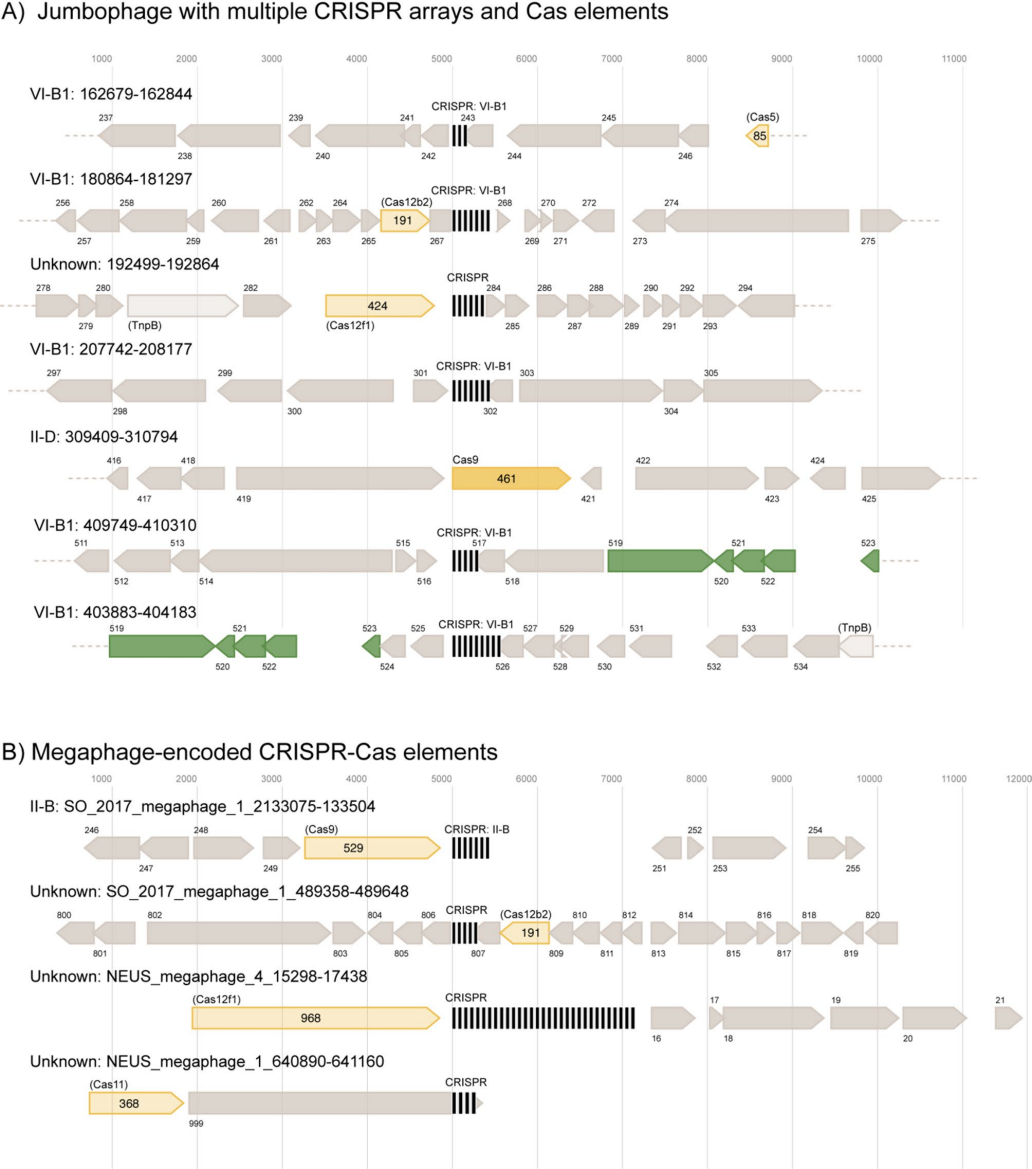
CRISPR in phage genomes of notable interest. **A**: CRISPR arrays and operons from a viral element that encodes six CRISPR systems and **B**: CRISPR array and operons from all CRISPR-encoding megaphages. Cas(-like) protein interference/effector modules are noted in yellow and CRISPR arrays are denoted by clusters of vertical black lines. Unknown genes are dark grey, and putative TnpB homologs are light grey. All predicted interference/effector modules were considered speculative annotations [50] and are indicated by reduced opacity and parentheses around the predicted protein name. Protein length in amino acids is indicated within each highlighted gene. The most closely related Cas subtype classification is noted in each case, followed by the coordinates of each CRISPR array on each phage genome. ORFs proximal to predicted CRISPR arrays, Cas elements, and TnpB-encoding genes are numbered in order of occurrence within a single genetic element. In some cases, CRISPR arrays/CRISPR-Cas operons and their surrounding regions overlap with one another, resulting in ORFs depicted more than once in the figure (highlighted in green). Phage hallmark genes and other genes unrelated to CRISPR-Cas systems are not annotated. This image was generated using CRISPRCasTyper and processed using Affinity Designer

The viral element with six CRISPR arrays is a predicted jumbo phage that is 421,673 bp in length and >95% complete, according to CheckV. All six arrays were confirmed using CRISPRCasTyper [50] and five of these arrays were confirmed with CRISPR-CasFinder [51], the latter of which assigns evidence levels of 1–4 (4 being the strongest) to its predictions. CRISPR-CasFinder predicted a total of 9 CRISPRs in this element, but 3/9 of those predictions were low confidence (evidence level 1) and were not identified by our workflow or CRISPRCasTyper, 1/9 had an evidence level of three and was not detected by our workflow or CRISPRCasTyper, and 5/9 had an evidence level of four and were detected both by our workflow and CRISPRCasTyper. CRISPRCasTyper also predicted a variety of CRISPR-Cas types and subtypes from this six-CRISPR-encoding viral element, which included the annotation of putative effector nucleases (Fig. 3A). Three of the megaphages (with genome sizes of 678, 641, and 508 kbp) were predicted to encode CRISPR systems (Fig. 3B). Two of these megaphages exceed the ~ 620 kbp size of the previously largest circularized CRISPR-encoding phage genome [34].

### Virally encoded class II effector nucleases

Viruses encode some of the most streamlined CRISPR-Cas systems identified to date [17, 34, 35]. Compact and efficient Cas effector nucleases are targets of interest for biotechnological applications. To identify putative streamlined Class II effector nucleases, a dataset of 1,494 ORFs ≥ 300 and ≤ 800 amino acids was identified from the 10 kbp regions upstream and downstream of the 515 putative virally encoded CRISPR arrays. DNA-targeting class II effectors were predicted from these ORFs using profile HMMs and blastp searches.

**Fig. 4:**
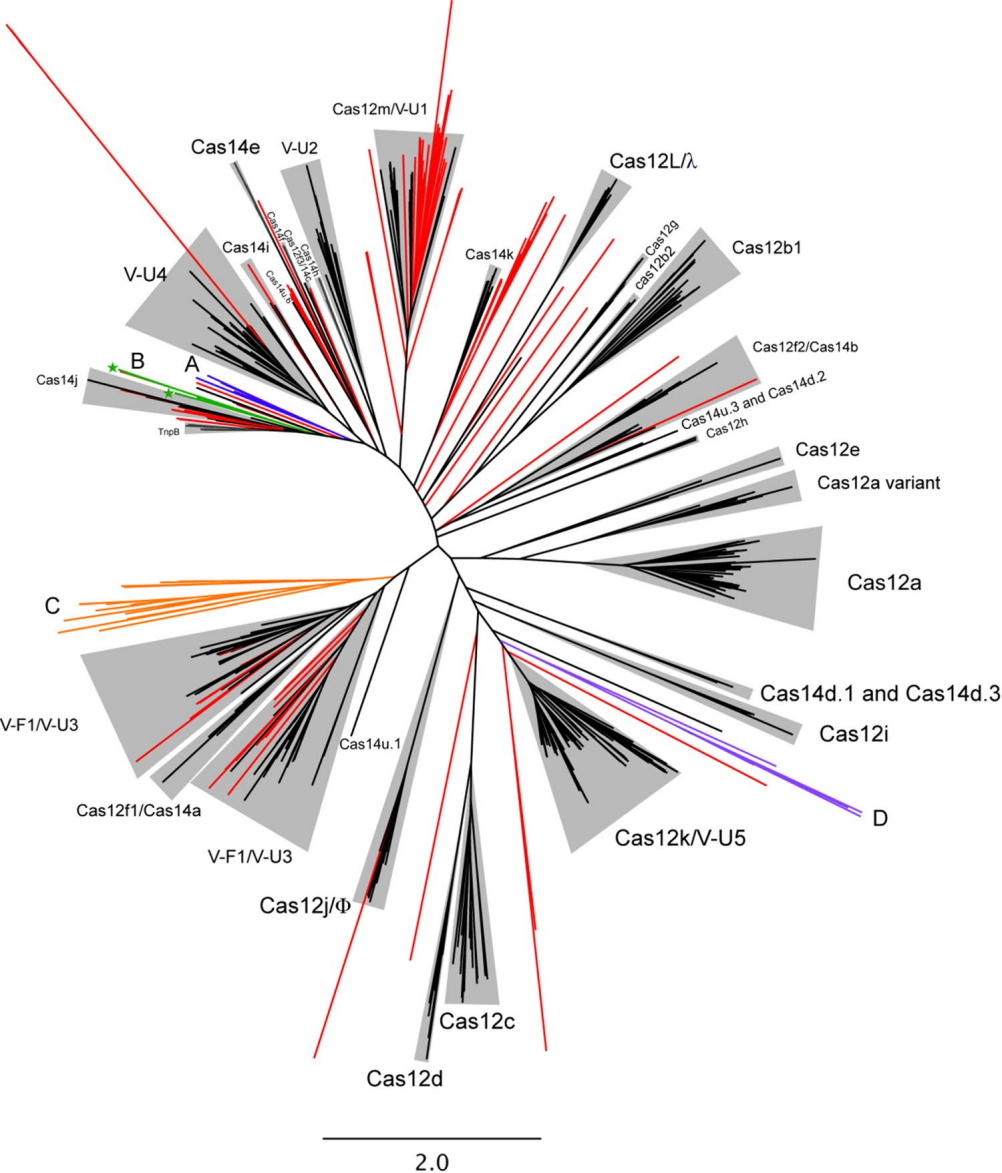
Type V CRISPR-associated nucleases, including virally encoded effectors from this study and reference nucleases. Putative nucleases from this study are coloured, where those in known groups or singletons are in red, and those in clades of further interest are denoted by a colour and a letter: A + blue, B + green, C + orange, and D + purple. Reference nucleases are in black. The two nucleases of specific interest (all three RuvC motifs, AlphaFold results in line with active nucleases) are marked with stars on the tree. Names assigned to clades are those of (putative) effector nucleases, regardless of whether they have been experimentally characterized. In cases where the Type V subtype of a predicted nuclease has been classified, but the predicted nuclease has not been assigned an official Cas protein name, the predicted effector nucleases (e.g., c2c9; [53]), is denoted in the tree by its subtype classification (e.g., VU-2). Legacy names of nucleases that have been renamed during reclassifications are included for clarity, as are the subtypes of recently characterized nucleases, even if they have received an official Cas protein name as nuclease/subtype (e.g., Cas12m/V-U1). Singletons and landfill-exclusive clusters not associated with a specific named clade are unlabeled

A total of 161 putative type V nuclease ORFs were identified after dereplication. These ORFs were placed in a reference tree that included known type V nuclease and TnpB sequences (Fig. 4). We observed no virally encoded nucleases that grouped with Cas12 subtypes a, b1, b2, c, d, e, f1, g, h, and i, likely because most of these protein families, with the exception of f1, b2, and g, have an average length that exceeds the 800 aa limit set for our ORFs of interest. The 800 aa limit was set to specifically target Cas protein effectors that are streamlined in size, i.e., smaller in size relative to the current most streamlined, experimentally-validated, virally encoded class II Cas effector, Cas12j/CasΦ, which are ~ 800 aa in size ([17, 35], see Methods for more details). We observed multiple nucleases that associated on the tree with other subtypes of Cas12 (i.e., f2 (1), f3 (1), j (1), and m (24)) and Cas14 (e (1), i (2), j (7), and k (9)), each of which average less than 800 aa in size per subtype. We examined each of these sequences for RuvC motifs, where the seven Cas14j-associated proteins contained all expected RuvC motifs and others were less readily confirmed (see Supplemental Results for more information). We also observed two predicted TnpB sequences encoded by viruses. These putative TnpB sequences have all three RuvC motifs of TnpB and are 401aa and 433aa in length, respectively, in line with TnpB lengths of 350–550 aa [52]. These sequences are both encoded proximal to CRISPR arrays, consistent with the hypothesis that TnpB associated with CRISPR multiple times to give rise to different type V CRISPR-Cas systems, and the observation that TnpB is frequently encoded by phages [34]. In total, 114/450 (25%) of viral elements that encode CRISPR arrays were predicted to encode a type V homolog, consistent with the recently-observed enrichment of these hypercompact systems in phage genomes [34] relative to bacterial and archaeal genomes [53]. Of these 114 viral elements, 10 (8.7%) are predicted to encode 2–3 unique type V effector proteins. One of these ten viral elements encodes three distinct predicted V-U4 nucleases, and all ten of these viral elements were predicted to encode at least one subtype V-U nuclease, or a nuclease that is most closely related to a V-U subtype nuclease. Lastly, we observed multiple nodes/clades on the type V phylogeny that only include sequences from our study (Fig. 4), representing additional unexplored novelty within this family that are interesting targets for nuclease activity characterization. None of the phages encoding the putative effectors highlighted on Fig. 4 were predicted to encode spacer acquisition machinery. This is consistent with previous studies showing that nearly all CRISPR-encoding phages lack adaptation machinery [17, 34].

### Potentially novel class II effector nucleases

All clades containing only sequences from this study on the Type V effector phylogeny (Fig. 4) were examined on a case-by-case basis for motifs of interest. We identified 6 sequences (300–399 aa) that grouped proximal, but distinct to Cas14j (Fig. 4, A/blue at 10 o’clock). These sequences had very weak RuvCI motifs, weak but stronger RuvCII motifs, and only the three longer sequences (385, 398, and 399 aa) had detectable RuvCIII motifs. A second cluster of 7 sequences (372–534 aa) was not associated with a specific Cas protein clade on the tree (Fig. 4, B/green, 9 o’clock). All 7 had all 3 detectable RuvC motifs (i.e., RuvC[I, II, III]), but only two sequences had strong signatures for all RuvC motifs. We identified a clade of 19 sequences (Fig. 4, C/orange, 8 o’clock) that, from sequence divergence, was predicted to be a new subtype of Type V effector protein, however, manual examination of these 19 sequences revealed no RuvC motifs. A final cluster of five sequences was identified proximal to the Cas12k/VU-5 clade (Fig. 4, D/purple, 4 o’clock), but only one of these sequences had a single RuvC motif, specifically RuvCIII. Numerous singleton predicted effectors were present on the tree, with most containing zero or only one identifiable RuvC motif (in all cases, RuvCIII).

### Structural examinations of potentially novel class II effector nucleases

We used AlphaFold3 (AF3) [53] to obtain structural predictions for our potentially novel Cas effectors of interest, i.e., those that were in distinct clades in our type V effector tree in which we were also able to detect all RuvC motifs (two sequences, 440 aa and 372 aa (both: Fig. 4, B/green, stars)). We also used AF3 to predict the structure of a potentially novel type V effector subtype (Fig. 4, C/orange, 8 o’clock, one representative sequence of 474 aa was selected). We also used AF3 to predict structures for the reference Cas effectors that were most closely related to our three predicted effectors (i.e., Cas14j), as well as all phage-encoded Cas effectors (Cas12L/Casλ and Cas12j/Φ).

Predicted template modelling (pTM) scores for the landfill sequences of interest were as follows: Sequence of interest 1 (440 aa): 0.8, Sequence of interest 2 (372 aa): 0.87, and Sequence of interest 3 (474 aa): 0.6. The most similar folds were between our sequences of interest and Cas14j. This was confirmed with US-align [54] which yielded the highest template modelling (TM) scores (max of 0.66) for the comparison of our sequences of interest and a representative Cas14j sequence (451 aa). TM-scores between our sequences of interest and Cas12L and Cas12j were ~ 0.4.

To further examine the similarities resulting in the TM-score of 0.66 (between Cas14j and Sequence of interest 2) we compared both structures using UCSF ChimeraX [55]. Root Mean Square Deviation (RMSD) between 174 pruned atom pairs was 1.226 Å (across all 363 pairs: 7.243 Å). For reference, between our Sequences of interest 1 and 2: RMSD between 310 pruned atom pairs was 0.900 Å; (across all 369 pairs: 2.845).

## Discussion

Here we examined the diversity of viruses across 27 locations within three municipal waste sites. Viral DNA represented 2–13.5.5% of the landfill metagenomes and depicted diverse viral communities. Limited overlap was observed between viral communities from our landfill samples and those from wastewater, groundwater, anaerobic digesters, and other environments. When we compared our landfill communities to each other using Vclust, we observed that the majority of our landfill viruses did not cluster across distinct landfills. This suggests that the landfill virome diversity is large, distinct relative to related ecosystems, and remains undersampled. Using geNomad, ~ 97% of landfill viral elements were classified at the class level, but only ~ 1.7% were classified at the order level (or family level, in cases where there was no assigned order), to a total of 15 orders and 12 families. The inability to classify most of our landfill viral elements beyond the class level is a clear indication of the lack of representation of these viruses in current reference databases. Landfill leachate viromes were more diverse and heterogeneous than those observed in a survey of upstream municipal waste treatment processes (waste storage, transfer, and treatment of organic waste), where the viromes were dominated by Microviridae in contrast to the enrichment in Caudoviricetes seen in our samples [56]. This indicates the various components of the waste management pipeline house different, novel viruses.

Across all landfill datasets, we identified a total of 138 jumbo phage genomes (i.e., those ≥ 200 kbp in size) with seven predicted to be complete or nearly complete megaphage genomes (i.e., ≥ 500 kbp). One of these putative megaphage genomes, SO_2017_megaphage_1, is ~ 678 kbp long, which is, to our knowledge, the third largest phage genome reported to date. It is noteworthy that the two largest megaphage genomes reported in literature (735 and 717 kbp) were described from reports that examined 13 ecosystems (including all data from the Tara Oceans project and Global Oceans Virome) [17], and 231 freshwater metagenomes [44], respectively – these reports were also published over three years apart. This suggests that bacteriophage genomes of this size are very rare in nature, making seven megaphage genomes from only two ecosystems (landfills and their adjacent groundwater wells) an unexpected discovery. The number of genes encoded by the seven megaphages ranges from 643 to 1,128, with 10–20 predicted AMGs per genome (Table S4). Three of the seven megaphages encode CRISPR arrays, with the 641 kbp and 678 kbp phage genomes now being the largest reported phage genomes to encode CRISPR arrays (exceeding the previous length of 620 kbp [34]). None of the seven megaphages were targeted by MAGs within our virus-host networks, nor did the CRISPR arrays encoded by these viruses target any other viruses in our data. We were able to identify the large terminase subunit genes for six of the megaphages, which we used to place them in a reference tree of huge phages (Fig. 1). Four of our megaphage genomes > 600 kbp grouped together in a clade that included some of the largest phage genomes described in literature [17, 44], all of which are provisionally classified as “Mahaphages” and have previously been predicted to infect members of the phylum Bacteroidetes [17, 44]. None of the landfill Mahaphage were connected to a host in our survey. This suggests that while huge phages are taxonomically diverse [17], the phages with the largest genomes have shared ancestry across unconnected environments.

In our virus-host networks, as seen in our previous work [7], we observed hyper-targeted viral elements and viral elements predicted to infect across distinct phyla. Our consistent observation of putative cross-phylum infecting viruses in landfills, despite this phenomenon being reported from a limited number of other studies [1, 8–10], suggests that landfills may be enriched for these broadly infecting viruses. Recent work showed that these very broad host range viruses are most often observed in syntrophic biofilms found in densely populated ecosystems [10]. Landfills are typically anaerobic systems where labile carbon is utilized fully through microbial handoffs and syntrophy, as evidenced by consistent and long-term methanogenesis [57]. Syntrophy is predicted to be widespread in landfills [57], and biofilm modes of life likely dominate in solid waste repositories, meaning the ecosystem characteristics of landfills are consistent with those predicted to select for broadly infecting viruses [10]. While we manually curated our putative cross-phylum interactions, and what we observe is consistent with existing literature, we note that because we solely utilize CRISPR spacer-viral protospacer matches to identify these interactions, these observations could stem from incorrectly binned CRISPR arrays. In-vitro evidence is necessary to substantiate these predictions.

We identified conserved CRISPR spacers across our different landfill samples to test whether landfills with higher levels of virus-host cross-targeting between sites also shared similar protospacer targets in viral genomes. Consistent with our examinations of viral cross-targeting, both SO_2016 and SO_2017 shared higher proportions of spacers with NEUS_2019 compared to any pairing with CA_2019 (Table 5). CA_2019 was also the most distinct site in the Vclust clustering of the landfill viral datasets, so all three lines of evidence support that it houses a less conserved viral community compared to the other two landfills. Omitting shared spacers between SO_2016 and SO_2017, as they are temporal datasets from the same landfill, ~ 1–3% of spacers were shared between SO samples and NEUS_2019 with 100% identity and equal length. This suggests that landfills that share more similar viruses are more likely to share host CRISPR spacers, however, these shared spacers represent a small fraction of the spacer complement at a site, and do not account for all cross-targeting observed between these sites.

From a total of 106,364 putative viral elements, 450 (0.42%) were predicted to encode CRISPR arrays, a subset that has an average length of ~ 48 kbp. These observations are remarkably consistent with a previous study examining virally encoded CRISPR systems in which 0.4% of phages encoded CRISPR-Cas systems and had an average length of 52 kbp [34]. CRISPR-encoding viruses in this study did not show any infection bias to a particular host taxon.

A jumbo phage with an approximately 421 kbp genome was predicted to encode six CRISPR arrays, five of which were annotated by two state-of-the-art CRISPR prediction tools [50, 51]. According to CRISPRCasTyper, only four of these six CRISPR arrays were proximal (i.e., within 5 kbp upstream or downstream) to any *cas* gene or gene predicted to encode a TnpB-like protein. In total, this jumbo phage contains genes predicted to encode two TnpB-like proteins, one Cas9, one Cas12b2-like protein, and one Cas12f1-like protein (Fig. 3A). The predicted *cas9* gene was not proximal to any CRISPR arrays (Fig. 3A). The disjunction of arrays and *cas* genes is potentially due to the guide RNAs from the two orphan CRISPR arrays directing effectors encoded elsewhere in the viral genome, or in the host genome [58]. The orphan arrays could also be explained by the presence of proximal effector genes that are too divergent to be detected by current models of *cas* genes. The potential presence of multiple class II effector encoding genes in a single viral genome, along with possible TnpB encoding genes, which may themselves be hypercompact DNA targeting systems [59], could aid these elements’ defense against Cas protein inhibitory systems, such as anti-CRISPR [60]. Since CRISPR-Cas systems also have roles in processes such as gene expression and signal transduction [61], and since viral CRISPR-Cas systems can target genetic systems of the host organism that regulate immunity [29] and host gene regulation [17], it is possible that the expansive CRISPR-Cas repertoire of viruses with large genomes may allow these phage to exert greater control over their host organism during infection, resulting in the creation of a “virocell” [62, 63].

We detected putative virally encoded effector proteins that clustered with multiple subtypes of Cas12 (i.e., f2, f3, j, and m), and Cas14 (e, i, j, and k), but most of our virally encoded type V-like effector proteins were located proximal to or with TnpB, the phage encoded systems Cas14j and Cas14k, and type V-U1, V-U5, and V-F1/VU-3 systems. All of these systems are either among the most streamlined type V or the progenitor (i.e., TnpB) systems. The subtype V-U systems are predicted as intermediates between TnpB and full-fledged type V effectors [53]. It is thus possible that viruses act as vectors for these intermediate systems, allowing an evolutionary path distinct from host-encoded systems, and with the potential to develop unique systems nearly exclusive to mobile genetic elements (MGEs) such as phages [17, 34], and to MGE-associated systems such as Tn7-like transposons [32, 64]. We identified 18 clades comprised of sequences exclusively from our study that were substantially divergent from previously described Type V effectors (Fig. 4). Some of these proteins may represent novel type V nucleases, with highest likelihood being the seven for which we were able to identify all RuvC motifs. Strong similarities in protein folds were observed between our proteins of interest and Cas14j proteins, which were also proximal to each other within our type V tree (Fig. 4). However, the overall structures of our three proteins of interest are distinct relative to reference Cas proteins and they are highly streamlined (440, 372, and 474 aa), which could confer advantages for biotechnological applications. The full or partial absence of identified RuvC motifs in the other proteins could be due to substantial divergence of these motifs from those in reference enzymes, as is the case for phage-encoded Cas12L [34], or absence of canonical catalytic sites within the RuvC domain, as is the case for Cas12m [65]. Absent or divergent RuvC motifs do not necessarily indicate loss of function or alternate function for these Type V effector-related proteins. Even if these catalytic motifs are entirely absent, these proteins may still demonstrate interference activity through binding to, but not cleaving, their target nucleic acid, a function that has been seen for dCas9 [66] and Cas12m [65]. Both dCas9 and Cas12m have been repurposed as biotechnological tools, as alternate interactions with nucleic acids beyond targeted cleavage have downstream uses. For the putatively novel effector nucleases in this study to be considered as biotechnological tools, they would need to be validated in vivo for interference and/or cleavage activities.

## Conclusions

In summary, landfill viral diversity is highly variable across landfills, and distinct relative to similar environment types such as anaerobic digesters, wastewater, and groundwater. Some of these viruses are predicted to broadly infect across the bacterial domain, which is consistent with recent, but limited literature. Bacteriophage genomes uncovered in this work are among the largest identified to date, sharing common evolutionary origins with the largest phage genomes described in literature. The discovery of seven of these phages from only 27 landfill metagenomes is in stark contrast to limited detection of these viruses in targeted studies that explored hundreds of metagenomes [17, 44]. Landfills may be a hot spot for discovering more of these megaphages, which may aid our understanding of the evolution of these phages and expand our knowledge of microbial genes that have been co-opted by giant phages. AMGs detected in this study have limited instances reported in literature, including genes involved in organochlorine and methane metabolism, both of which are relevant to landfills as contaminated sites that are globally significant producers of methane [37]. The discovery of AMGs in landfills that are relevant to landfill function but have rarely been observed elsewhere supports that genes mobilized within a microbial community are influenced by the selective pressures imposed by the environment.

We identified over 400 CRISPR-encoding viral elements from samples taken from three municipal landfills across North America. In some cases, these virally encoded CRISPRs were flanked by progenitor or full-fledged type V effector-like nucleases, including potentially novel branches of this protein family identified for the first time within this study. All of the putative type V effectors we identified in this study had a maximum length of 800 amino acids. If proven functional, these nucleases would help circumvent the size restrictions of certain viral delivery vectors such as those of Adeno-associated Viruses (AAVs) [67].

### Methods

#### Sampling

Sampling of the Southern Ontario (SO) landfill occurred twice one year apart (July 2016 [SO_2016] and October 2017 [SO_2017]) and has been described previously [7, 36]. Nine distinct cells of the NEUS landfill were sampled in February 2019 as described previously [37]. The final four metagenomes came from a closed landfill from Southern California (CA_2019) that was sampled in June of 2019. Leachate from one leachate well, the influent leachate to the leachate treatment plant, and liquid from the leachate treatment plant biofilter were collected in sterile 500 mL containers. All samples were passed through 0.2 μm Sterivex filters (Sigma Aldrich) until the filters clogged, with a minimum of three filters taken per sample (~ 50–500 mL of volume processed per site). The leachate treatment plant biofilter filter clogged very heavily. As a result, the sample was split into two for DNA extractions: the filter, and the solids filling the Sterivex filter cartridge. All landfill samples are detailed in Table S1.

The viral component of the SO landfill metagenomes was previously described from the perspective of viral-host interactions [7]. None of the other metagenomes have been analyzed for their viral components, and all cross-site comparisons as well as AMGs and megaphage analyses are novel to the current study.

#### DNA extractions, sequencing, and host genome binning

DNA extractions for the SO landfill and adjacent aquifers and the NEUS samples have been previously described [7, 36, 37]. DNA extractions for the CA landfill were completed using the Qiagen Power Soil Pro kit following the manufacturer’s instructions with the addition of the sterivex filter directly into the bead beating tube in place of soil. No viral enrichments were performed for any samples described in this study, i.e., all viruses were detected from sequencing DNA extracted from the 0.2 μm filters. The CA landfill samples underwent metagenomic sequencing at The Center for Applied Genomics (Toronto), generating 2 × 150 bp reads on an Illumina HiSeq. For metagenome assembly and host genome binning, the same pipeline as for previously analyzed landfill metagenomes was used. In brief, read trimming was accomplished with bbduk (https://github.com/BioInfoTools/BBMap/blob/master/sh/bbduk.sh) and sickle (https://github.com/najoshi/sickle). Scaffolds were assembled using Spades3 [68] (https://cab.spbu.ru/software/spades/) under the -meta flag with kmers 33, 55, 77, 99, and 127. Scaffolds ≥ 2.5 kbp were retained for further analyses. Read datasets were mapped to assembled metagenomes using Bowtie2 [69]. Scaffolds were binned using CONCOCT [70], MaxBin2 [71], MetaBAT2 [72], and consensus bins determined using DAS Tool [73]. Resultant bins were quality assessed using CheckM [74], and taxonomically classified using the Genome Taxonomy Database Toolkit [75]. Genome bins that were >70% complete and less than 10% contaminated (defined as, at minimum, medium quality draft MAGs [76] were used as putative host MAGs in further analyses. Metagenome sequencing, analysis, and host genome binning were previously described for the SO and NEUS samples [7, 37] but followed this same workflow from read quality control onwards.

#### Viral identification, classification, and genome binning

All described viral analyses were performed individually for each sample. All scaffolds with a minimum length of 5 kbp were analyzed with VirSorter2 [38] and CheckV [39] sequentially, following the protocol at 10.17504/protocols.io.bwm5pc86. Since CheckV, along with all other state-of-the-art tools, may not detect proviral region boundaries accurately, all proviruses identified by CheckV were discarded to maintain the integrity of further analyses, including AMG identification. All putative non-integrated viruses that had either at least one viral gene annotated by CheckV, or had zero viral and host genes annotated by CheckV, were retained for subsequent clustering with CD-HIT-est [40] using a global sequence identity threshold of c = 0.95. Viral genome binning was performed on the dereplicated set of viral scaffolds using vRhyme [41], and only vMAGs with ≤ 5 redundant proteins according to vRhyme were retained for subsequent analyses. To compare the composition of the landfill virome across landfills and to the composition of viromes from related and disparate ecosystems, we used Vclust [45] (with an average nucleotide identity threshold of 0.95) to cluster our ≥ 5 kbp landfill viral elements with 100,000 ≥ 5 kbp viral elements from anaerobic digesters, groundwater, and wastewater, respectively (each obtained from IMG/VR v4 [46]) and 100,000 ≥ 5 kbp viral elements sampled at random from IMG/VR v4. Taxonomic classification of predicted viral elements ≥ 10kbp was performed using geNomad’s [48] annotate module and geNomad database v1.5.

In cases where we identified putative megaphages, read mapping was used to confirm genome circularization across the direct terminal repeat regions. We annotated terminase large subunit ORFs using HMMER3 [77] and Hidden Markov Models (HMMs) from the Virus Orthologous Groups database (https://vogdb.org/, release number vog218) and the Prokaryotic Virus Orthologous Groups (pVOGs) database [78]. Predicted large terminase subunits were confirmed with VIBRANT [79]. Predicted large terminase subunit sequences were aligned with other giant phage large terminase subunit sequences [17] using MUSCLE [80] version 5.1.linux64 and the Super5 algorithm. ModelTest-NG [81] was used to predict the best protein evolutionary model from the MUSCLE alignment. A phylogenetic tree was inferred using RAxML v.8.2.12 [82] under the PROTGAMMAVTF model with automatic bootstopping for bootstrap support, and was visualized using Geneious [83].

#### Virus-host linking

Viral elements that were at least 10 kbp in size were linked to their hosts through matching host CRISPR array spacers to viral protospacers. CRISPR arrays in host MAGs were detected using MINCED v. 0.4.2 (https://github.com/ctSkennerton/minced) with the following parameters: -minSL [minimum spacer length] set to 25 and the -spacers flag included to output a fasta file of all identified CRISPR spacers. These CRISPR spacers were BLASTn [84] searched against viral elements with -task set as ‘blastn-short’, with alignments retained only if they had at most one mismatch, no gaps, a query coverage of ≥ 90% [2] and an E-value ≤ 10^−4^.

#### Cross-phylum infection curation

All host MAGs involved in putative cross-phylum interactions were manually examined to assess the legitimacy of the interaction and to reduce the likelihood of incorrectly binned scaffolds leading to predicted cross-phylum interactions. First, the CRISPR-array encoding scaffolds of the MAGs predicted to target a putative cross-phylum infecting virus were ensured to be at least 10 kb in length. Next, we required all MAGs predicted to be involved in cross-phylum interactions to have a maximum contamination of 5.00% according to CheckM.

#### Assessment of CRISPR-Cas targeting space within and across landfills

Using the same workflow described above in “*Virus-host linking*,” we examined how often host CRISPR spacers from a site targeted viral elements from the same site and from different sites. We next detected spacers that were encoded multiple times in a single dataset or those that were shared between data sets, with no mismatches. Reverse complements were considered matches, but hits between a query spacer and a subsequence of a larger spacer were not considered as matches, as an exact length match requirement was implemented.

#### Auxiliary metabolic gene detection

AMGs were detected by inputting all dereplicated viral scaffolds into VIBRANT, which provides AMG predictions, with the ‘--virome’ flag. Predicted AMGs with limited or no prior representation in literature were considered high interest AMGs and manually curated. The genomic neighborhoods of these putative AMGs were examined for obvious host genes, flanking viral hallmark genes, and hypothetical viral genes, to assess whether predicted AMGs were due to microbial scaffolds misannotated as viral scaffolds or microbial genes proximal to integrated prophage. The following criteria were used: (1) AMGs located at either end of a scaffold were filtered out. (2) AMGs that had a KEGG v-score [85] or Pfam v-score ≥ 1 were filtered out. (3) AMGs with flanking genes (four genes upstream or downstream) having a KEGG v-score < 0.25 were filtered out. To compare the incidence of high interest municipal waste site-derived AMGs to other systems, the 100,000 ≥ 5 kbp viral elements that were randomly sampled from IMG/VR v4 (described in Methods section “*Viral identification*,* classification*,* and genome binning”*) were subjected to the same AMG detection pipeline.

Phylogenetic trees were made for virally encoded alpha-amylase, coenzyme P450 hydrogenase subunit beta (FrhB), and RuBisCO large subunit (RbcL). For each protein, reference sequences were identified through best blastp hits (5–10 sequences selected, covering a broad taxonomic range), as well as from UniProtKB (FrhB, RbcL) or literature reviews of the protein family (alpha amylase). Alignments were generated using Muscle v. 3.8.425 [80] and masked to remove columns with more than 90% gaps in the Geneious [83]. ModelTest-NG [81] was used to predict the best protein evolutionary model from the MUSCLE alignments. Phylogenetic trees were inferred using RAxML v.8.2.12 [82] and were visualized using Geneious. Blastp and Foldseek [86] were used to confirm domain-level annotations for high-interest AMGs.

#### Viruses encoding CRISPR arrays

All predicted viral elements were screened for CRISPR arrays using MINCED v. 0.4.2 with the following parameters: -minSL [minimum spacer length] set to 25 and the -spacers flag included to output a fasta file of all identified CRISPR spacers. Interviral conflicts were predicted through BLASTn searching spacers from viral CRISPR arrays against.ffn files of Prodigal-predicted [87] ORFs from viral elements that did not encode CRISPR arrays (-task ‘blastn-short’, with alignments retained only if they had at most one mismatch, no gaps, a query coverage of ≥ 90% [2] and an E-value ≤ 10^−4^). We annotated hits using HMMs from the Virus Orthologous Groups Database (VOGDB) release 226 (https://vogdb.org/vogdb/226) and the corresponding.faa files from Prodigal.

#### Cas effector genes proximal to viral CRISPR arrays

CRISPRCasTyper [50] was used for automated detection of CRISPR-Cas operons in viral elements predicted to encode CRISPR arrays. Manual detection of Class II effector nucleases was performed as follows. All 10 kbp regions directly upstream or downstream from CRISPR arrays were examined for Open Reading Frames (ORFs) using Prodigal [87] (flags -m -p meta -q). All predicted ORFs that were 300–800 amino acids (aa) in length were retained for further analyses to identify streamlined Class II proteins. This 800 aa limit was set to target Cas protein effectors that were smaller in size than the current most streamlined, experimentally-validated, virally encoded class II Cas effector, CasΦ/Cas12j, whose members are ~ 800 aa in size [17, 35]. Class II effector-encoding *cas* genes proximal to CRISPR arrays were predicted by querying CRISPR-array-adjacent ORFs using profile Hidden Markov Models (HMMs) [88] obtained from previous studies [50, 89] and HMMER v.3.2.1 [77]. Additionally, BLASTp [84] searches were performed using multiple protein families that have been previously classified, and in some cases experimentally validated, as queries: TnpB (the predicted ancestor of Cas12 nucleases [53, 90]); subtypes of Cas12; V-U nucleases (some of which have not been validated for interference activity); and Cas14 [17, 53, 65, 91]; along with the recently identified and experimentally validated phage-encoded CasΦ (Cas12j) and Casλ (Cas12L). The screen did not include type VI effector proteins as queries, which are RNA-guided RNA targeting systems, as previous work demonstrates these systems are not nearly as common in viruses compared to the other Class II systems, i.e., type II and type V [34], and we were specifically interested in systems capable of DNA manipulation.

#### Identification of putatively novel Cas protein effectors

Examinations were focused on type II and type V nucleases, as diverse nucleases of these types were previously identified in phages [34]. All ORFs that hit to type II nucleases were manually examined for the presence of RuvC and HNH motifs. ORF hits to type V nucleases were gathered. Due to some type V nucleases having very low identity to other subtypes [34], we also generated a separate dataset of all ORFs proximal to viral CRISPR arrays that were not identified as matches using our profile HMM or BLASTp searches (“Proximal ORFs”). The Proximal ORFs were combined with all reference type V nucleases and TnpB sequences, and aligned using MUSCLE version 5.1.linux64 and the super5 algorithm. ModelTest-NG [81] was used to predict the best protein evolutionary model from the MUSCLE alignment. A phylogenetic tree inferred using RAxML v.8.2.12 [82] with -m PROTGAMMAVTF was visualized using Geneious. Since the Proximal ORFs/proteins that were not hit by reference HMMs could include *cas* genes that do not encode effector nucleases, or genes unrelated to CRISPR-Cas systems, putative effector proteins in this set were identified through BLASTp searching representatives of each clade of sequences on the phylogenetic tree against NCBI’s non redundant protein database (nr). A clade was retained only if the proteins/domains hit in nr were hypothetical proteins, TnpB, or endonucleases (excluding restriction endonucleases). Each retained clade was manually examined for RuvC motifs, and those with a detectable RuvC signature that also clustered close to known type V effector proteins and TnpB in the phylogenetic tree, were retained as putative Cas effector proteins. This subset of the Proximal ORFs dataset was then combined with the predicted effector ORFs that were identified through the HMM and/or BLASTp searches. This final aggregated dataset was aligned using MUSCLE version 5.1.linux64 and the PPP algorithm, due to smaller input size. ModelTest-NG [81] was used to predict the best protein evolutionary model from the MUSCLE alignment [80]. A phylogenetic tree was inferred using RAxML v.8.2.12 [82] with -m PROTGAMMAVTF and was visualized using Geneious (Fig. 4).

#### Structural examinations of putatively novel Cas protein effectors

AlphaFold3 (AF3) [92] was used to obtain structural predictions for our putative novel Cas effectors and reference Cas effectors. Structural comparisons were performed using US-align [54] to obtain template modelling (TM) scores. AF3 structures were further compared through examining root mean square deviation (RMSD) values obtained through superimposing putatively novel and reference Cas effector structures using UCSF ChimeraX’s [55] matchmaker function.

## Supplementary Information

Supplementary Material 1.

Supplementary Material 2.

## Data Availability

The datasets generated and/or analysed during the current study are publicly available as follows: A curated fasta file of all jumbo and mega phage identified in this study is available at the Open Science Framework under DOI 10.17605/OSF.IO/FQ46R. *The SO_2016 metagenomes* : The raw reads are available in the SRA database under accessions SRS2844134 (CLC1_T1), SRS2845087 (CLC1_T2), SRS2844583 (LW1), SRS2844135 (LW2), SRS2844137 (LW3), and SRS2844136 (GW1). Assembled and annotated scaffolds are available on the Integrated Microbial Genomes and Microbiomes (IMG) database with the following IMG Genome IDs (Taxon Object IDs): 3300014203 (CLC1_T1), 3300014206 (CLC1_T2), 3300014204 (LW1), 3300015214 (LW2), 3300014205 (LW3), and 3300014208 (GW1) (https://img.jgi.doe.gov/cgi-bin/m/main.cgi). All MAGs from SO_2016 and SO_2017 connected in virus-host networks are available on NCBI under the BioProject PRJNA823399 and accessions JALNVE000000000- JALOCK000000000. Scaffolds predicted to be entirely or partially viral are available in IMG, with all viral IMG accessions listed in Supplemental File 1. *The SO_2017 metagenomes*: The raw reads are available in the SRA database under accessions SRS4258091 (CLC), SRS12485771 (GW1), SRS4258089 (GW3), SRS12485774 (LW1), SRS4258090 (LW2), SRS12485775 (LW3), SRS4336822 (LW4), and SRS4258104 (SWC). The assembled and annotated SO_2017 metagenomes are deposited on IMG with the following IMG Genome IDs (Taxon Object IDs): 3300028602 (CLC), 3300037810 (GW1), 3300028028 (GW3), 3300035208 (LW1), 3300028603 (LW2), 3300037067 (LW3), 3300029288 (LW4), and 3300028032 (SWC). All MAGs from SO_2016 and SO_2017 connected in virus-host networks are available on NCBI under the BioProject PRJNA823399 and accessions JALNVE000000000- JALOCK000000000. Scaffolds predicted to be entirely or partially viral are available in IMG, with all viral IMG accessions listed in Supplemental File 1. *The NEUS metagenomes* are housed under BioProject PRJNA900590. Within this BioProject, the raw reads files are available in the SRA database, under Biosamples SAMN31696084 – SAMN31696092. The 1,892 MAGs are available in the WGS database under accessions SAMN32731718 – SAMN32731810 (STA), SAMN32731811 – SAMN32731998 (STB), SAMN32733587 – SAMN32733720 (STC), SAMN32734194 – SAMN32734413 (STD1), SAMN32734415 – SAMN32734683 (STD2), SAMN32734737 – SAMN32734946 (STE), SAMN32737191 – SAMN32737484 (STF1), SAMN32737485 – SAMN32737723 (STF2). Viral scaffolds are available in the WGS database under accessions JBINNZ000000000-JBINOH000000000. *The CA_2019 metagenomes* are housed under BioProject PRJNA1070006. The reads are available in the SRA database under accessions SRR27751815-SRR27751818. The MAGs implicated in viral-host interactions are available under accessions JBFWIF000000000-JBFXLM000000000. Viral scaffolds are available in the WGS database under accessions JBINOI000000000 - JBINOL000000000.
