# Supplementary material for: Discarded diversity: novel megaphages, auxiliary metabolic genes, and virally encoded CRISPR-Cas systems in landfills
Source: Virol J. 2025 Nov 11;22:370. doi: 10.1186/s12985-025-02990-6 (PMC12607003; doi:10.1186/s12985-025-02990-6)
Supplement: Supplementary file 2 — Supplementary Material 2. [file 12985_2025_2990_MOESM2_ESM.pdf]

Discarded diversity: Novel megaphages, auxiliary metabolic genes, and virally encoded CRISPR-Cas systems in landfills

Nikhil A. George[1], Zhichao Zhou[2], Karthik Anantharaman[2], Laura A. Hug[1,*]

[1] – Department of Biology, University of Waterloo, Waterloo ON, Canada

[2] – Department of Bacteriology, University of Wisconsin – Madison, Madison, WI, USA

NAG: nageorge@uwaterloo.ca

ZZ: zczhou2017@gmail.com

KA: karthik@bact.wisc.edu

LAH: laura.hug@uwaterloo.ca, * corresponding author

Supplemental Materials

Table of Contents:

Supplemental Results p. 2-3

Supplemental Tables p. 4-5

Supplemental Figures p. 6-8

Supplemental Results

Similarity to previously identified virally encoded CRISPR-Cas systems

We observed several instances where our predicted effector sequences were clustered with or were most closely related to previously identified virally encoded CRISPR-Cas systems (i.e., Cas14j (7), Cas14k (9), Cas14i (2), Cas12j (1), and Cas12L (1), Figure 4), of which only Cas12j and Cas12L have been experimentally validated for function [1,2]. Each of our relevant sequences was examined for RuvC motifs and assessed for similarity to the virally encoded nuclease it clustered most closely with.

Seven sequences ranging from 373-441 aa clustered with previously identified Cas14j sequences (378-451aa; Figure 4, 10 o'clock). Our sequences showed high sequence similarity to Cas14j sequences and contained all three RuvC motifs (RuvCI-III). Six additional sequences clustered proximal but distinct to the Cas14J cluster and are described in the main text in more detail. We detected two sequences of lengths 402 and 509 aa that clustered with Cas14i (Figure 4, 11 o'clock). Both sequences, as well as reference Cas14i proteins, had detectable RuvCI and RuvCIII motifs but very weak, if detectable, RuvCII motifs. Nine sequences ranging from 324-512 aa clustered proximal to previously identified Cas14k sequences (Figure 4, 1 o'clock). All nine sequences contained RuvCI motifs, two contained RuvCII motifs, and four contained RuvCIII motifs. Only two of these nine sequences (of lengths 410 and 422 aa) contained all three RuvC motifs. We detected one sequence clustering with Cas12j (Figure 4, 7 o'clock). This sequence lacked the RuvCIII motif, as did Cas12j6, one of the 10 reference Cas12j nucleases [3]. Our putative Cas12j sequence was also missing key residues in the RuvCII domain, which Cas12j6 lacked entirely, and key residues in the RuvCI motif, all of which were present in Cas12j6. Only three orthologs of Cas12j(1-3) have been experimentally confirmed for function [2]. The comparisons made to Cas12j6 add confidence to the assignment of our query sequence

as a Cas12j ortholog, despite its lack of key catalytic residues. Our putative Cas12j is the shortest within the clade, at 346 aa compared to 441 (Cas12j6 from a giant phage [3]) and 708-813aa for the remaining 9 Cas12j proteins. While our sequence branches within the Cas12j clade, its activity is less confidently predicted based on the aberrant characteristics described above. Notably, our putative Cas12j sequence is encoded by a predicted plasmid, the second time a Cas12j-like protein was identified on a plasmid [4].

Supplemental Tables and Figures

Table S1: Landfill sites and sampling details

Site	Sample ID	Sample type	Metagenome size (Gbp)	BioSample Accession	SRA Accession
SO_2016	LW1	Leachate well	26.58	SAMN07630781	SRX3574636
	LW2	Leachate well	30.00	SAMN07630782	SRX3574178
	LW3	Leachate well	29.98	SAMN07630780	SRX3574180
	CLC1_T1	Composite leachate cistern	29.89	SAMN07630778	SRX3574177
	CLC1_T2	Composite leachate cistern	28.16	SAMN07630777	SRX3575198
	GW1	Groundwater well	25.58	SAMN07630779	SRX3574179
SO_2017	LW1	Leachate well	15.57	SAMN27259107	SRX14723681
	LW2	Leachate well	40.52	SAMN10350574	SRX5256784
	LW3	Leachate well	47.58	SAMN27259106	SRX14723680
	LW4	Leachate well	38.75	SAMN10863920	SRX5344198
	CLC	Composite leachate cistern	38.72	SAMN10350766	SRX5256785
	SWC	Storm water catchment	21.34	SAMN10350495	SRX5256798
	GW1	Groundwater well	51.22	SAMN27259105	SRX14723679
	GW3	Groundwater well	18.76	SAMN10350765	SRX5256783
NEUS	A	Leachate well	53.99	SAMN31696084	SRX18288880
	B	Leachate well	47.93	SAMN31696085	SRX18288881
	C	Leachate well	56.03	SAMN31696086	SRX18288882
	D1	Leachate well	52.88	SAMN31696087	SRX18288883
	D2	Leachate well	48.60	SAMN31696088	SRX18288884
	E	Leachate well	37.64	SAMN31696089	SRX18288885
	F1	Leachate well	57.67	SAMN31696090	SRX18288886
	F2	Leachate well	50.17	SAMN31696091	SRX18288887
	CSWMC	Composite leachate cistern	54.34	SAMN31696092	SRX18288888
CA_2019	LW1	Leachate well	31.10	SAMN39634476	SRX23416964
	CLC	Composite leachate cistern	64.38	SAMN39634477	SRX23416965
	TP_BF	Treatment plant biofilter - planktonic	61.73	SAMN39634478	SRX23416966
	TP_BS	Treatment plant biofilter - solids	58.01	SAMN39634479	SRX23416967

Table S2: Putative cross-phylum host-virus interactions.

Sample set	Putative hosts	Host MAG phylum (GTDB-tk)	Host completion and contamination (%)	# host spacer to viral protospacer matches	Predicted viral element
CA_2019	TPIn_75<br>TPBF_198	Desulfobacterota<br>Proteobacteria	99.41, 0.00<br>90.75, 0.63	1<br>7	vMAG_518
NEUS_ 2019	STF2_137<br>STCSWMC_88	Bacteroidota<br>Firmicutes_A	95.56, 3.26<br>80.02, 4.08	1<br>2	vMAG_1257
NEUS_ 2019	STCSWMC_93<br>STF1_64<br>STF2_19<br>STD2_245<br>STCSWMC_50<br>STCSWMC_25	Bacteroidota<br>Bacteroidota<br>Bacteroidota<br>Bacteroidota<br>Cloacimonadota<br>Firmicutes_B	94.35, 0.27<br>95.43, 0.27<br>95.43, 0.00<br>88.71, 2.42<br>98.90, 2.20<br>90.15, 4.60	15<br>16<br>1<br>5<br>1<br>3	vMAG_3146
NEUS_ 2019	STF2_137<br>STCSWMC_88	Bacteroidota<br>Firmicutes_A	95.56, 3.26<br>80.02, 4.08	1<br>2	vMAG_910
NEUS_ 2019	STF2_144<br>STCSWMC_50<br>STF2_148<br>STCSWMC_25	Cloacimonadota<br>Cloacimonadota<br>Cloacimonadota<br>Firmicutes_B	100.00, 1.10<br>98.90, 2.20<br>95.54, 1.10<br>90.15, 4.60	1<br>2<br>2<br>2	NODE_3233_length_30680_cov_384 .260596_NEUS_F2||full
SO_2017	LW2_137<br>LW2_139	Muirbacteria<br>Patescibacteria	93.26, 4.56<br>70.53, 3.61	2<br>4	vMAG_2310

Tables S3 and S4 are included as a single .xlsx file "Supplementary File 1.xlsx"

Table S3: Predicted AMGs encoded across all datasets.
Table S4: AMGs encoded by megaphage genomes.

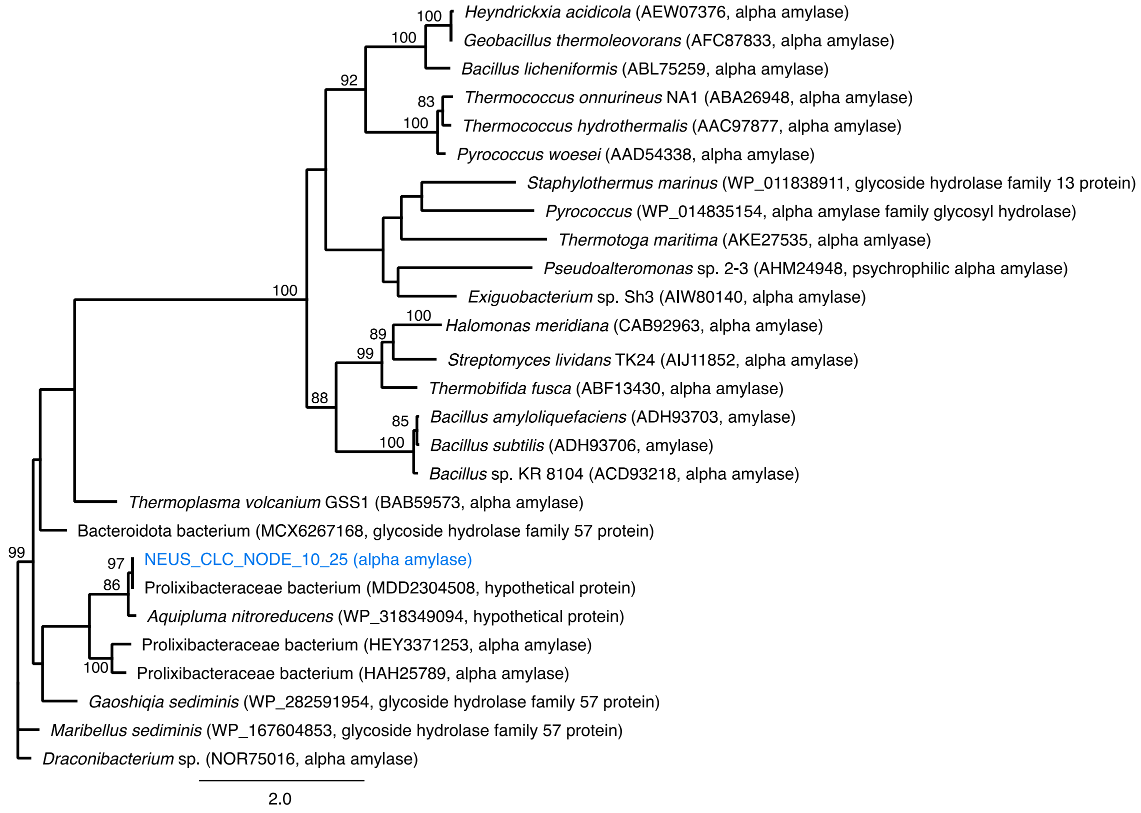

Figure S1: A maximum likelihood phylogeny of the alpha amylase AMG (blue), including representative best hits from a blastp [5] search and reference sequences [6]. The final alignment contained 28 taxa and 805 unambiguously aligned columns. Alignments were generated with Muscle version 3.8.425 [7] and trimmed to remove columns with more than 90% gaps. The tree was generated using RAxML version 8 under the VT+I+G model of evolution [8] and visualized in Geneious [9].

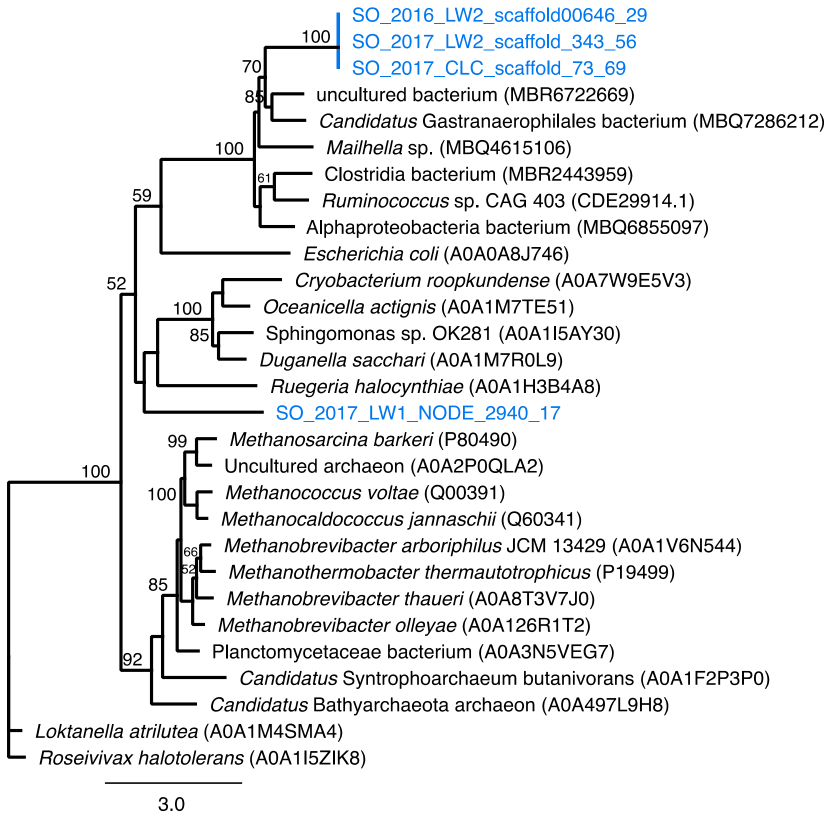

Figure S2: A maximum likelihood phylogeny of the coenzyme P450 hydrolase subunit beta AMGs (blue), including representative best hits from a blastp [5] search and reference sequences from UniProtKB [10]. The final alignment contained 29 taxa and 845 unambiguously aligned columns. Alignments were generated with Muscle version 3.8.425 [7] and trimmed to remove columns with more than 90% gaps. The tree was generated using RAxML version 8 under the LG+I+G model of evolution [8] and visualized in Geneious [9].

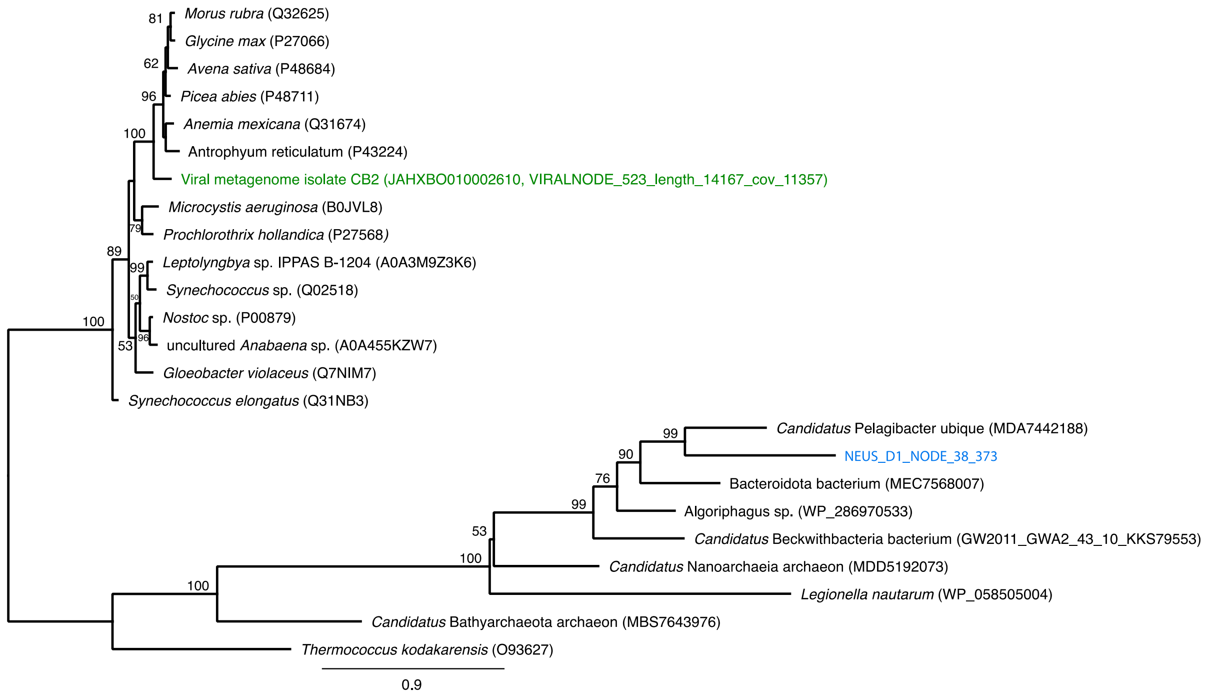

Figure S3: A maximum likelihood phylogeny of the RuBisCO large subunit AMG (blue), including representative best hits from a blastp [5] search and reference sequences from UniProtKB [10]. One previously reported RbcL from a viral fragment was also included (green, [11]). The final alignment contained 24 taxa and 481 unambiguously aligned columns. Alignments were generated with Muscle version 3.8.425 [7] and trimmed to remove columns with more than 90% gaps. The tree was generated using RAxML version 8 under the LG+I+G model of evolution [8] and visualized in Geneious [9].

References

1. Al-Shayeb B, Skopintsev P, Soczek KM, Stahl EC, Li Z, Groover E, et al. Diverse virus-encoded CRISPR-Cas systems include streamlined genome editors. Cell. 2022;185:4574-4586.e16.

2. Pausch P, Al-Shayeb B, Bisom-Rapp E, Tsuchida CA, Li Z, Cress BF, et al. CRISPR-CasΦ from huge phages is a hypercompact genome editor. Science. 2020;369:333–7.

3. Al-Shayeb B, Sachdeva R, Chen L-X, Ward F, Munk P, Devoto A, et al. Clades of huge phages from across Earth's ecosystems. Nature. 2020;578:425–31.

4. Pinilla-Redondo R, Russel J, Mayo-Muñoz D, Shah SA, Garrett RA, Nesme J, et al. CRISPR-Cas systems are widespread accessory elements across bacterial and archaeal plasmids. Nucleic Acids Res. 2022;50:4315–28.

5. Altschul SF, Gish W, Miller W, Myers EW, Lipman DJ. Basic local alignment search tool. J Mol Biol. 1990;215:403–10.

6. Mehta D, Satyanarayana T. Bacterial and archaeal α-Amylases: diversity and amelioration of the desirable characteristics for industrial applications. Front Microbiol. 2016;7:1129.

7. Edgar RC. MUSCLE: multiple sequence alignment with high accuracy and high throughput. Nucleic Acids Res. 2004;32:1792–7.

8. Stamatakis A. RAxML version 8: a tool for phylogenetic analysis and post-analysis of large phylogenies. Bioinformatics. 2014;30:1312–3.

9. Kearse M, Moir R, Wilson A, Stones-Havas S, Cheung M, Sturrock S, et al. Geneious Basic: An integrated and extendable desktop software platform for the organization and analysis of sequence data. Bioinformatics. 2012;28:1647–9.

10. The UniProt Consortium. UniProt: the Universal Protein knowledgebase in 2023. Nucleic Acids Res. 2023;51:D523–31.

11. Bhattarai B, Bhattacharjee AS, Coutinho FH, Goel RK. Viruses and their interactions with Bacteria and Archaea of hypersaline Great Salt Lake. Front Microbiol. 2021;12:701414.
